# Dispersal or Drift? More to Plant Biodiversity Than Meets the Eye

**DOI:** 10.1371/journal.pbio.0030043

**Published:** 2005-01-04

Over 250 million years ago (mya), all the continents of Earth formed a single land mass called Pangaea. Some 50 million years later, this supercontinent began to split in two, forming Laurasia—now North America, Asia, and Europe—and Gondwana—present-day Antarctica, Australia, South America, Africa, and India. After another 50 million years, Gondwana, too, broke up. At the end of the Cretaceous period, New Zealand split off (about 80 mya), then South America and Australia separated from Antarctica (about 35 mya). Fairy-tale quality aside, the story of continental drift fits comfortably with the geological and fossil record and feeds our understanding of current distributions of plant biodiversity.

Although we know how and when Pangaea broke apart, the distribution of fossils of the same species on many different continents, separated by vast ocean waters, challenges us to explain how they got there. Plant life on New Zealand, for example, shares striking similarities to that on other Southern Hemisphere land masses, but scientists have yet to agree on how this came to pass. In particular, one genus, Nothofagus—the southern beech tree, a plant whose 80-million-year-old fossil history goes back to the days of Gondwana—has polarized views on the nature of Southern Hemisphere biogeography.

One theory suggests that geographic barriers (New Zealand and Australia are separated by the Tasman Sea) would have prevented species expansion after the break-up of the continents, so similar contemporary species must have already existed in both places before New Zealand broke away from Gondwana. In this scenario, called vicariance, ancestors of existing lineages drifted with the repositioned land masses. Another hypothesis, born of existing distributions and fossil data, suggests that long-range oceanic dispersal is more likely. But since Nothofagus seeds are not considered ocean-worthy vessels, many believe vicariance is the only possible explanation.

Peter Lockhart and colleagues argue that a clear picture of the divergence dates of various southern beech species could help clarify the relative contributions of vicariance versus dispersal. But they would need significant lengths of DNA sequences to reliably characterize the evolutionary history of each species.

Consequently, Lockhart and colleagues analyzed a 7.2-kilobase fragment of the chloroplast genome (which typically ranges from 110,000 bp to 160,000 bp) for 11 species of three Nothofagus subgenera—Lophozonia, Fuscospora, and Nothofagus—from South America, Australia, and New Zealand. Reconstructing the trees' evolutionary relationships (phylogeny) based on analyses of their chloroplast sequences, the authors discovered a nuanced evolutionary history that supports vicariance for some species and dispersal for others.[Fig pbio-0030043-g001]

**Figure pbio-0030043-g001:**
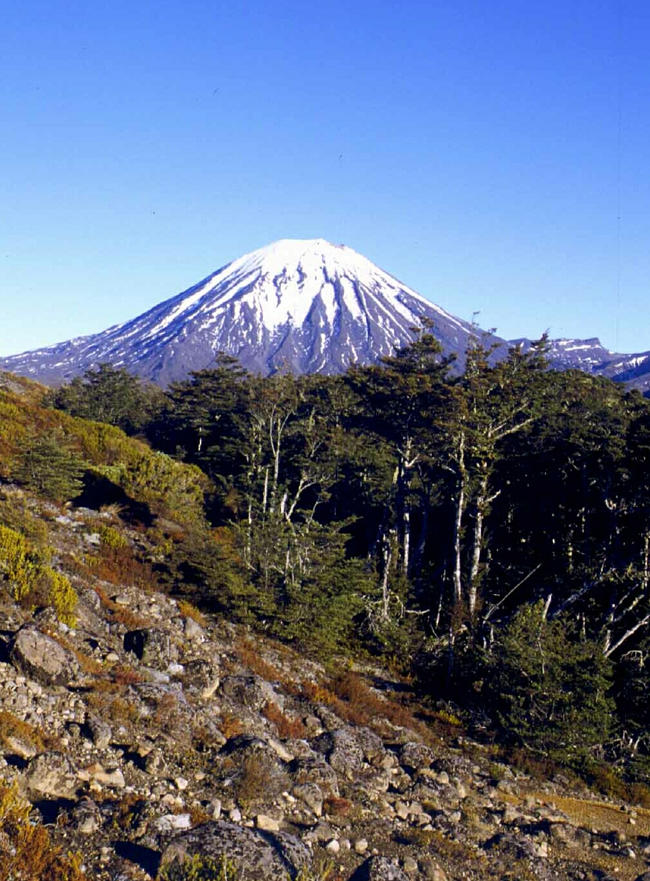
Nothofagus, the southern beech, on the slopes of Mt. Ruapehu in New Zealand (Photo: Peter Lockhart)

Assuming that beech was present throughout Gondwana (which fossil data support), the sequence of the Gondwana breakup should be reflected in the beech's phylogeny. New Zealand beeches should be more distantly related to both Australian and South American species, because of the greater period of separation—65 million years compared to 30 million years. Yet Australian and New Zealand beeches are more closely related to each other than to South American species, which reflects more recent relationships. Given that fossils of all beech subgenera extend back to the New Zealand Cretaceous period, the dating of splits and the nature of the relationships indicate extinction of beech lineages within current subgenera in New Zealand, and possibly in Australia and South America.

Lockhart and colleagues' analyses suggest that the relationships of the Australian and New Zealand Lophozonia and Fuscospora species are too recent to have roots in Gondwana, indicating a role for transoceanic dispersal. The evolutionary relationship between the Australasian and South American Fuscospora lineages, however, is consistent with vicariance. These divergence results, the authors conclude, indicate that current distributions of Nothofagus cannot be explained solely by continental drift (followed by extinction of some species) and that contemporary New Zealand Nothofagus species are not direct descendants of the beeches thought to have reached the island after the split from Antarctica.

Taken together, the results highlight the need for caution in evaluating fossil evidence. The fossil record doesn't necessarily capture when a species first appeared, and a continuous fossil presence can mask extinctions and reinvasions. The authors conclude that their molecular data make the case for investigating possible mechanisms of long-range dispersal—especially the dispersal properties of Nothofagus seeds—and stresses the need to consider more complex hypotheses to explain something as dynamic and complex as the evolutionary history of biodiversity.

